# Supplementary material for: Stable choice coding in rat frontal orienting fields across model-predicted changes of mind
Source: Nat Commun. 2022 Jun 10;13:3235. doi: 10.1038/s41467-022-30736-3 (PMC9187710; doi:10.1038/s41467-022-30736-3)
Supplement: Supplementary file 1 — Supplementary Information [file 41467_2022_30736_MOESM1_ESM.pdf]

Supplementary Information

Stable choice coding in rat frontal orienting fields across model-predicted changes of mind

J Tyler Boyd-Meredith*, Alex T Piet*, Emily Jane Dennis,
Ahmed El Hady+ & Carlos D Brody+

Contents

1 Individual Rat Behavior **2**
Accumulation model parameters 2
All rats' psychometric curves 3
All rat's chronometric curves 4
All rat's psychophysical kernels 5

2 Electrophysiology **6**
Recording locations and types 6
Example cell rasters 6
Selective cell population average PSTH 6

3 Supplementary Methods **8**
Accumulation model posterior distribution 8
Toy model demonstration 9
Verification of accumulation model posterior solution 9

4 Accumulation value tuning curves **13**
Rank 1 decomposition variance explained 13

5 Supplementary Note 1 **14**
Model State Changes 14

1 Individual Rat Behavior

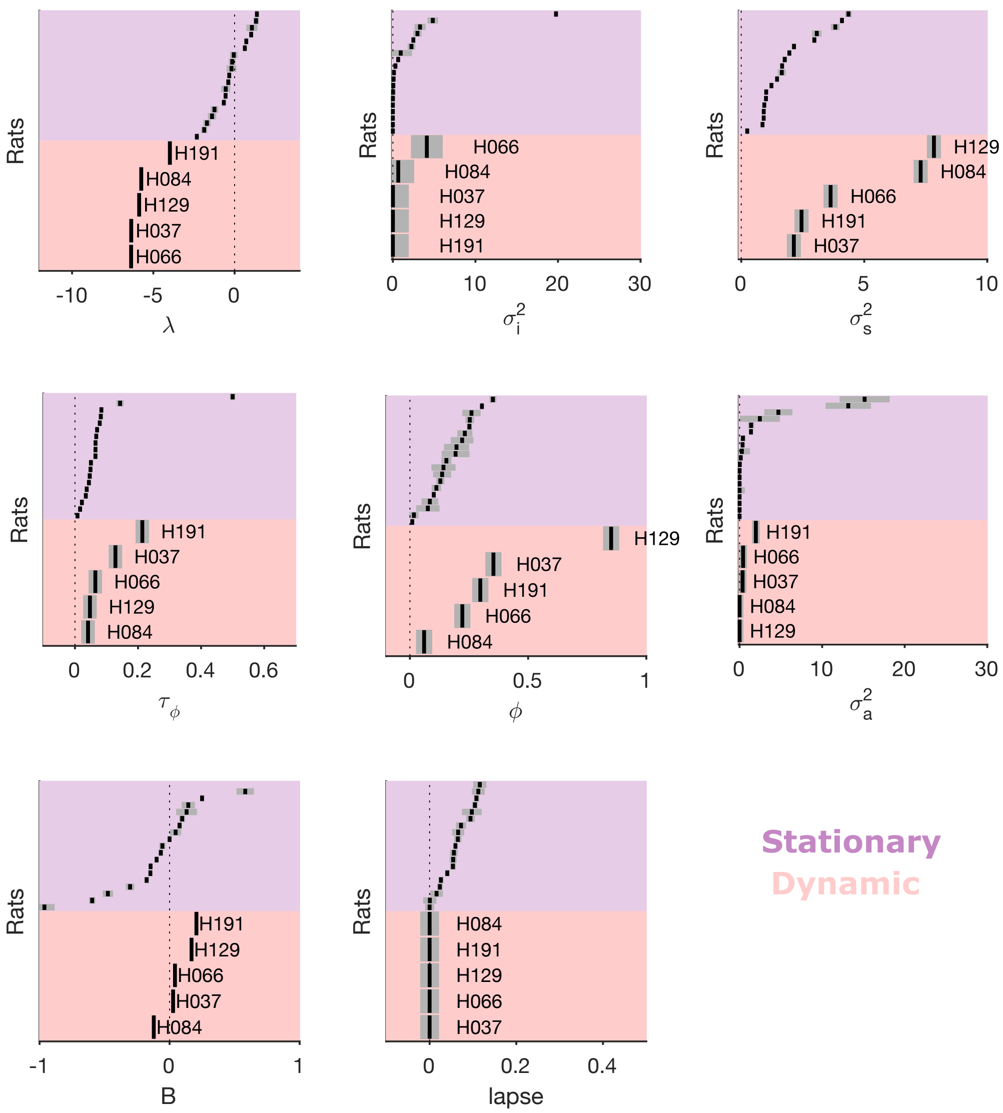

Supplementary Figure 1: **Accumulation model parameters** Best fit model parameters and 95% confidence intervals for each rat in this study. In addition the model parameter fits reported in[1] for 19 rats in a stationary environment are included for comparison. Brunton *et al.*,[1] also included sticky (absorbing) evidence boundaries as an additional parameter. Consistent with[2], we did not include this absorbing parameter because it was previously found not to improve model fits[1] and because the large discounting rate λ prevents the boundaries from influencing the model. The difference in lapse parameters may be explained by the large evidence discounting rates λ, the lack of absorbing bounds in the model, or simply a difference in rat behavior. Source data are provided as a Source Data file.

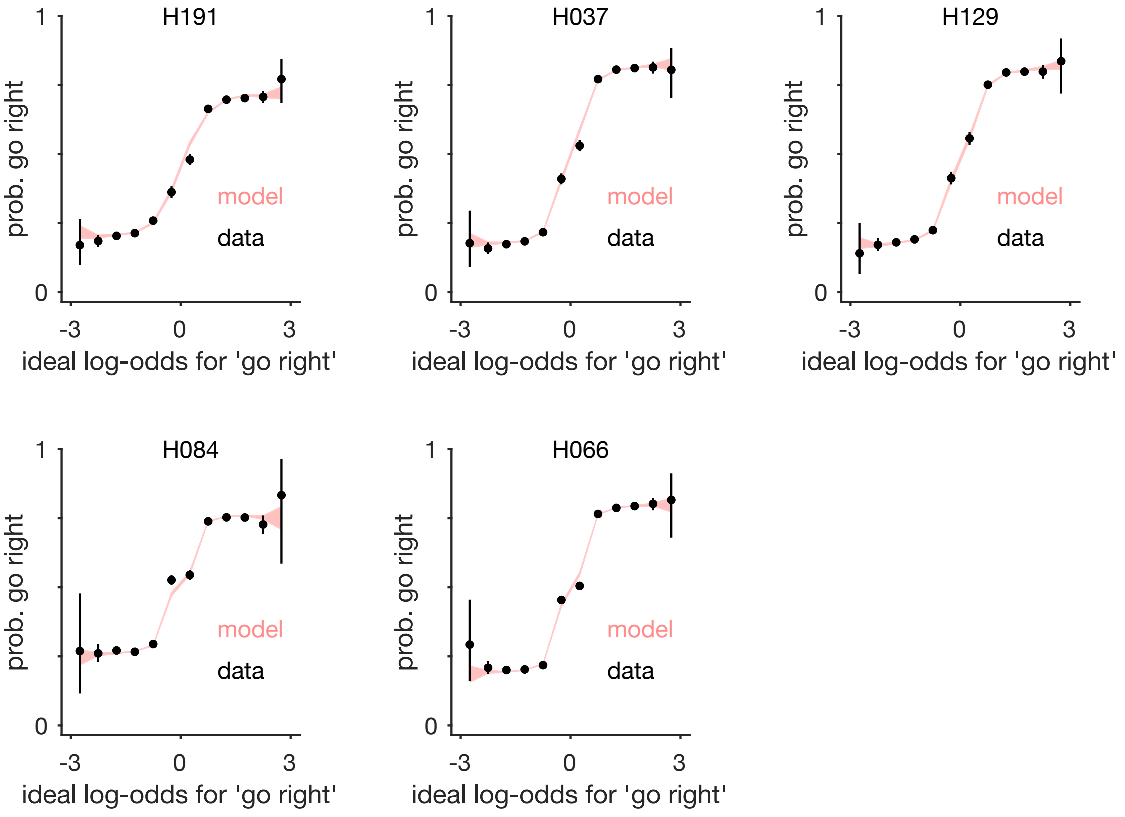

Supplementary Figure 2: **All rats' psychometric curves** Each plot shows the probability of a right choice given an ideal observer's log-odds supporting a go-right choice. Rat data (black points) is overlaid on predictions of the accumulation model with parameters fit to this rat (red traces). Errorbars for rat data represent 95% binomial confidence intervals around the mean (n=78,300/189 trials/sessions for H191; n=92,468/252 for H037; n=63,494/118 for H129; n=121,276/249 for H084; n=185,091/308 for H066). Source data are provided as a Source Data file.

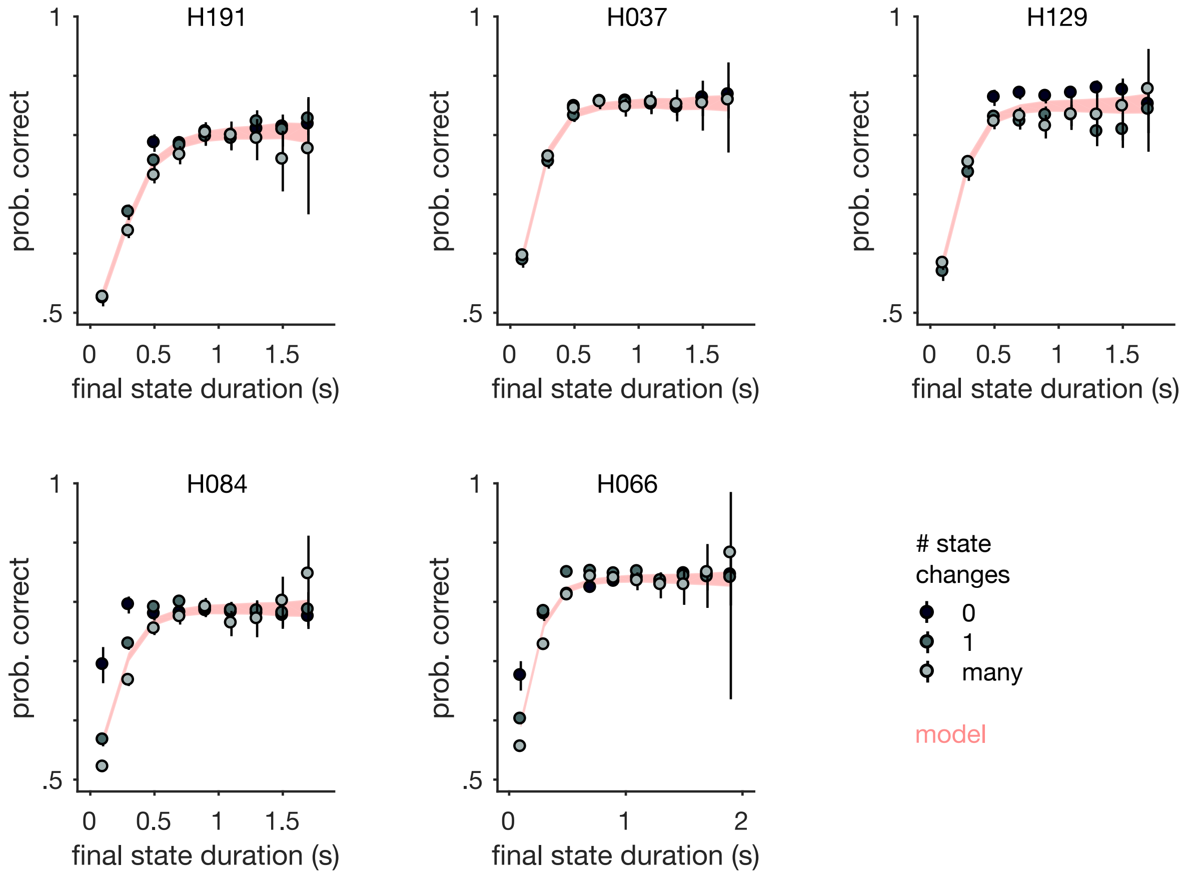

Supplementary Figure 3: **All rats' final state chronometric curves** Each plot shows the probability the rat made the correct choice (mean with 95% binomial confidence intervals) as a function of the final state duration, and the total number of state changes in the trial. The final state duration was defined as the time from the last environmental state change to the end of the stimulus period. The best fit model prediction averaged over the total number of state state changes is shown in red. Datasets are the same as in Fig. 2. Source data are provided as a Source Data file.

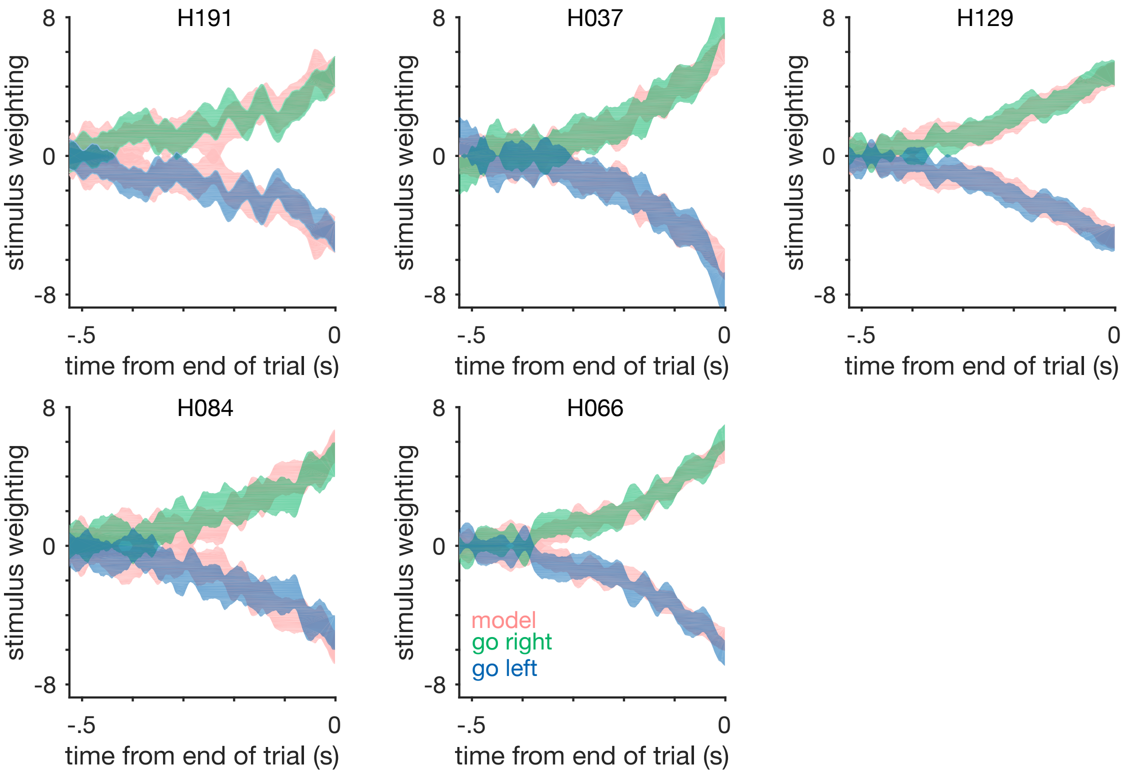

Supplementary Figure 4: **All rats' psychophysical reverse correlation kernels** Each plot shows the reverse correlation kernel for the rat (blue/green) and the best fit model (red). Green and blue patches indicate strength (mean ± s.d.) of evidence favoring rightward choice as a function of time until the trial ends for rightward and leftward choices, respectively. Datasets are the same as in Fig. 2 and 3. Source data are provided as a Source Data file.

2 Electrophysiology

Rat	Implant Location	Recording Type
H191	blFOF	tetrode
H037	rFOF	array
H129	lFOF	array
H084	lFOF	array
H066	rFOF	array

Supplementary Table 1: List of rats used in the study with recording location and acquisition method.

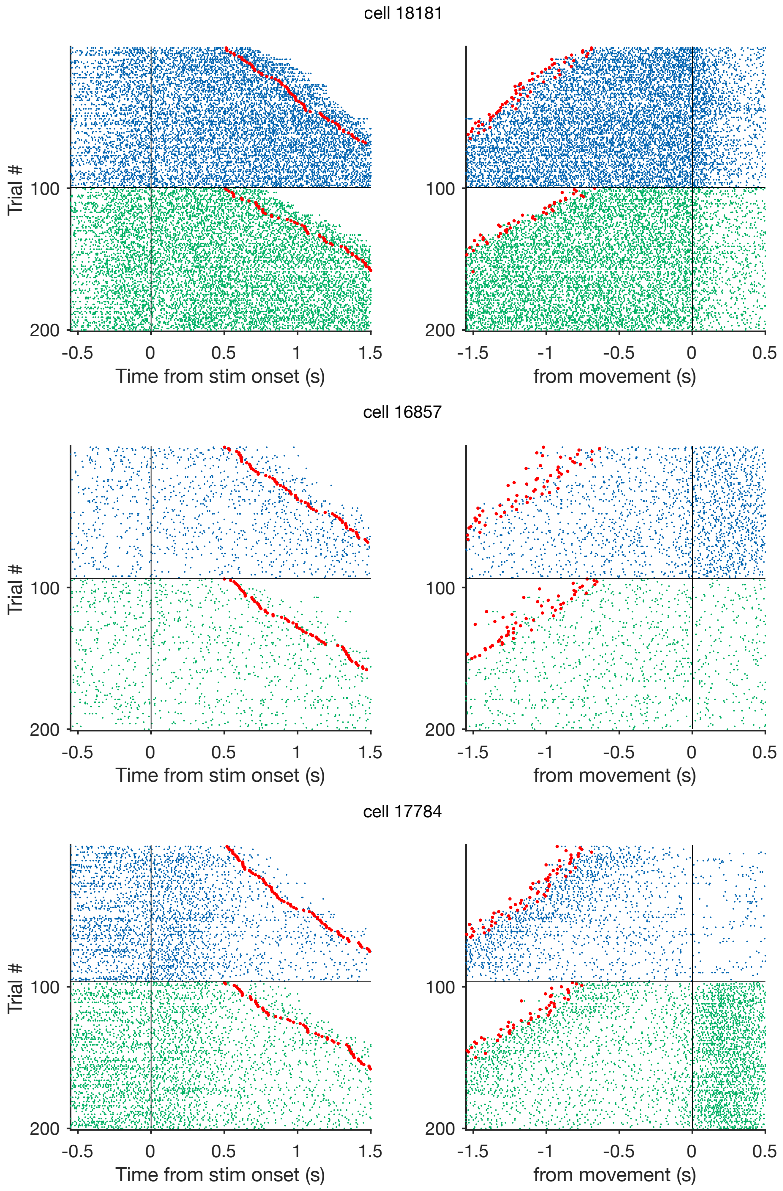

Supplementary Figure 5: **Example cell rasters** Trials are sorted by side-choice and stimulus duration. Trials are randomly subsampled to include 200 trials for each cell. Spikes are colored by side-choice (blue for left choices; green for right choices). Red points mark the end (left) or beginning (right) of the stimulus period. Source data are provided as a Source Data file.

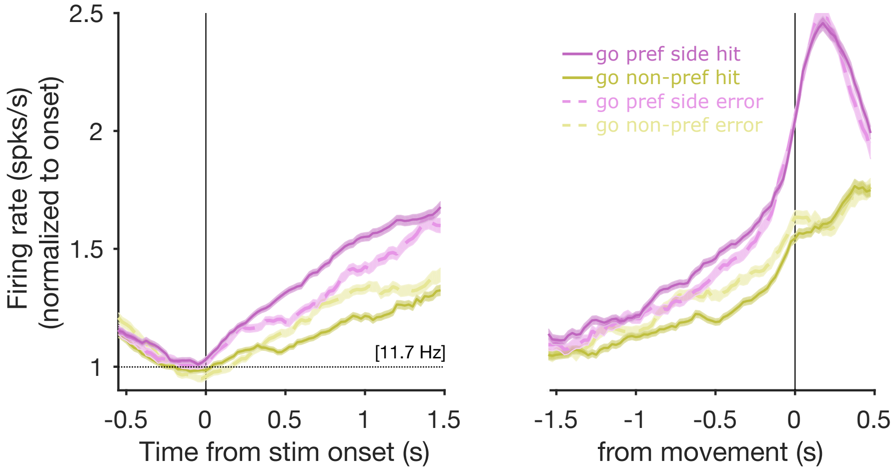

Supplementary Figure 6: **Selective cell population average PSTH** Average activity of all pre-movement side-selective cells (n=103) conditioned on side choice, outcome and cell preference. Purple traces represent choices of the cells' preferred side and yellow traces represent choices of the cells' non-preferred side. Dark, solid traces represent correct choices and light, dashed traces represent errors. Shaded regions represent s.e.m. Source data are provided as a Source Data file.

3 Supplementary Methods

Accumulation model posterior distribution

To compute the accumulation model posterior distribution used for neural data analysis (referred to as the "backward pass distribution" in[1]), we first found the parameter set θ that best explained each rat's choices y under the forward model $f(a)$ by maximizing $P(y|\theta)$, per equation 16 of the main text. Then, we use the best fit parameter set to evaluate the forward model for each trial, producing a probability distribution over accumulated evidence value at each time point given the initial accumulation value $a_0 \sim \mathcal{N}(0, \sigma_i^2)$. The posterior distribution $p(a)$ combines this constraint on initial conditions with a constraint on the final conditions given by the rat's choice, using a backward distribution $b(a)$ to impose the constraint on the final conditions of the accumulation value distribution. The backward model makes no assumptions about the initial conditions, but instead constrains the final conditions to be consistent with the rat's choice at the end of the trial t_N.

$$b(a) = P(a|t, \delta_R, \delta_L, \theta, y) \tag{1}$$

$$= \begin{cases} P(a|t, \delta_R, \delta_L, \theta, a_N \geq B), & \text{if } y = 1 \\ P(a|t, \delta_R, \delta_L, \theta, a_N \leq B), & \text{if } y = -1. \end{cases} \tag{2}$$

Importantly, the forward and backward distributions are conditionally independent, conditioned on the final value of the accumulated evidence. Given that they are independent, the posterior distribution that combines both observations is the product of the forward and backward distributions.

$$p(a) = P\left(a|t, \delta_R, \delta_L, \theta, a_0 \sim \mathcal{N}(0, \sigma_i^2), y\right) \tag{3}$$

$$\propto f(a)b(a) \tag{4}$$

It is important to note here that the adaptation process C is deterministic, and its evolution does not depend on the stochastic per-click noise realizations. We can consider this as an upstream sensory adaptation that happens before the integration process. As a result, the adapted clicks can be included in the forward and backward distributions.

One technical wrinkle is that our analytical solution for the model relies on initial conditions that are Gaussian. This presents no problem for the forward model, which assumes a Gaussian initial distribution $a_0 \sim \mathcal{N}(0, \sigma_i^2)$. However, when we compute the backward model, we begin at the final time point, where the final accumulation value is constrained to be on the side of the decision boundary corresponding to the animal's choice:

$$a_N \geq B, \text{ if } y = 1 \tag{5}$$

$$a_N \leq B, \text{ if } y = -1. \tag{6}$$

From this final time point, the model evolves backwards in time in response to the adapted clicks. This constraint on the final accumulation value can be thought of as a constraint on the initial conditions of the backward model, presenting a problem for our solution's dependence on Gaussian initial conditions. To solve this, we constructed a solution by discretizing the a-value axis into small bins of width Δa, and solved the backward model for each bin assuming a delta function of initial probability mass in each bin. We refer to the backward distribution from

each bin j as the delta-backward solution $b_j(a)$. Our entire backward distribution is the mixture distribution over all the individual delta-backward solutions.

$$b(a) = \sum_{j=0}^{\pm\infty} w_j b_j(a) \tag{7}$$

$$b_j(a) = P(a|t, \delta_R, \delta_L, \theta, a_N \sim \mathcal{N}(B + j\Delta a, 0)) \tag{8}$$

To solve each delta-backward solution $b_j(a)$, we use the time-reversed solution to the forward model. The mixture weights w_j are all equal if the bin spacing is uniform. Note that it might be tempting to think that we need to weight each individual delta-backward solution by the forward model's probability mass in each bin; however, this is not correct. Given that the backward model is independent of the forward model, we want the complete backward distribution to reflect all possible states consistent with the choice observation, which is the uniform distribution over the correct sign of a. With a set of $b_j(a)$ solutions, we can now combine them into the posterior distribution, $p(a)$. The exact solution as $\Delta a \to 0$ is given by:

$$p(a|t, \delta_R, \delta_L, \theta, a_0 \sim \mathcal{N}\left(0, \sigma_i^2\right), y) \propto f(a) \sum_{j=0}^{\pm\infty} w_j b_j(a). \tag{9}$$

In practice we truncate the infinite series at a suitable extrema value of a, and use a finite bin spacing $\Delta a = 1$. On each trial, the extent of the grid of delta solutions was determined by finding the accumulation value where less than $1e-4$ probability mass of the model posterior lay beyond that point at the end of the trial. Given that $f(a)$, and $b_j(a)$ will be Gaussian, let $p_j(a) = f(a)b_j(a)$, which is also Gaussian. This lets us write the posterior distribution as the sum of many delta-posterior modes.

$$p(a|t, \delta_R, \delta_L, \theta, a_0 \sim \mathcal{N}\left(0, \sigma_i^2\right), y) \propto \sum_{j=0}^{\pm\infty} w_j p_j(a). \tag{10}$$

Toy model demonstration

To illustrate this solution to the model posterior, consider a simple random walk process. On each time step there is a 1/3 probability of staying in place, 1/3 probability of taking a step of size +1, and a 1/3 probability of taking a step of size −1. We start at $a(t = 0) = 0$, and then observe the process ten time steps later, $t = 10$. We now want to compute the forward model, and the posterior distribution for this data.

Using a binomial distribution we can analytically compute the forward model, which describes the probability of observing the process given the initial conditions and an elapsed duration (Fig. 7A). To compute a backward model, we must define the final value. For example, if we let the final value of the random walk be $a(t = 10) = 2$, then we can again use the binomial distribution to compute the distribution of possible values at earlier time steps (Fig. 7B). We can alternatively let the final value be the sign of the random walk process and compute the full backward distribution. Combining the independent forward and backward distributions, we can predict the posterior distribution, which is all possible states the random walk could be in given these two observations (Fig. 7E). We can check this against a particle simulation by sampling 2,000,000 trajectories from the random walk process to get the forward distribution (Fig. 7C). To get the posterior distribution we filter our samples for trajectories that ended up with positive value (Fig. 7F). We can compare these two distributions by taking a slice in time (Fig. 7D). We can now move on to the accumulation model, which has the same basic random walk structure, but with a few more bells and whistles.

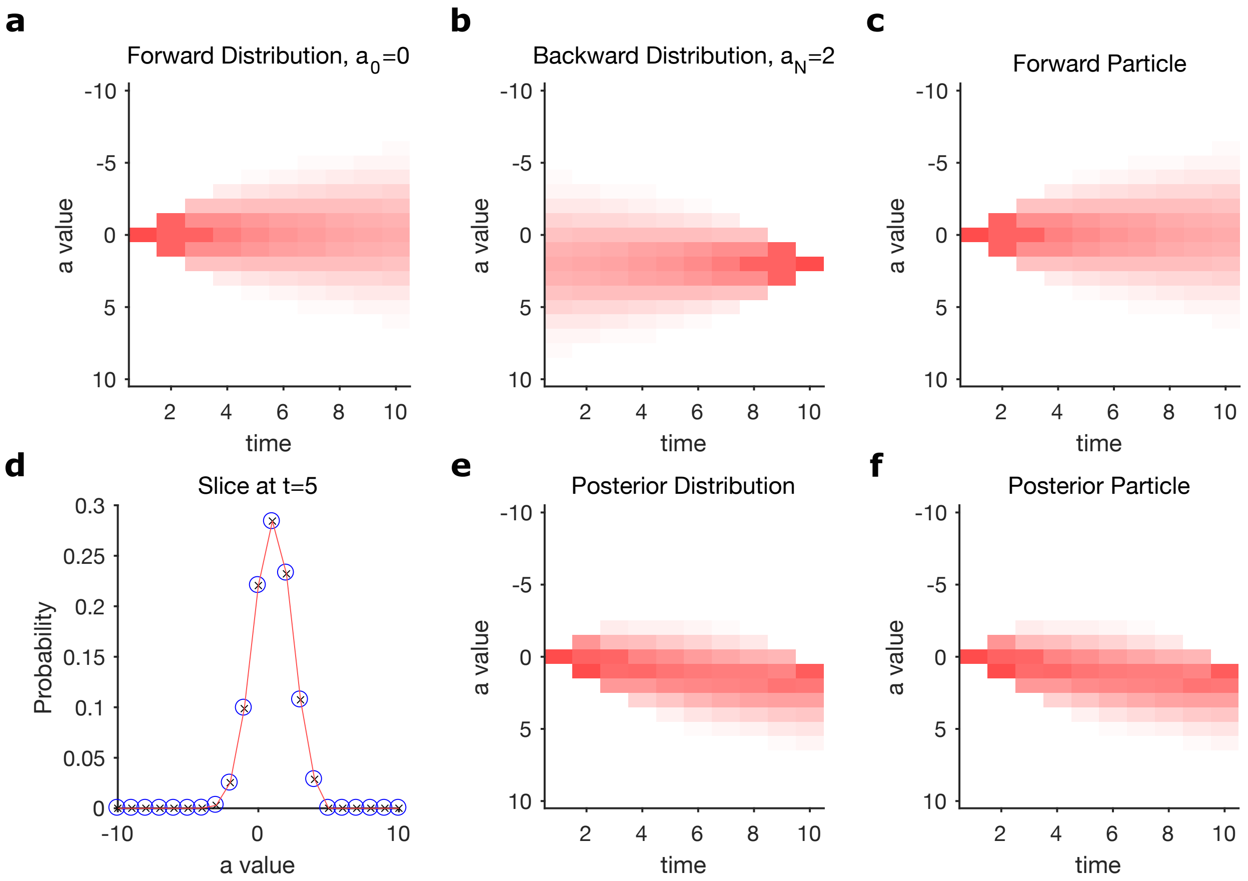

Supplementary Figure 7: **Toy model demonstration** (A) Forward model analytical solution for the toy model. (B) Delta-Backward model analytical solution for final accumulation value of $a_n = 2$. (C) Distribution of the forward model from sampling. (D) Comparing the distributions from E (red) and F (blue) at $t = 5$. (E) Model posterior analytical solution for initial condition $a_0 = 0$ and final conditions consistent with "go right" choice $a > 0$. (F) Model posterior solution from sampling. Source data are provided as a Source Data file.

Verification of accumulation model posterior solution

To illustrate the model posterior solution for the full accumulation model we can again compare the analytical solution to sampled trajectories, this time for an example trial. Each trajectory has a unique noise realization.

For both the model and sample trajectories, we have four distributions (Fig. 8). The forward distribution showing the predicted trajectories given the initial conditions. The backward-delta distribution showing the possible trajectories that result in a single final accumulation value. The posterior-delta distribution showing the possible trajectories that start at the initial condition, as in the forward distribution, and result in a single final accumulation value, as in the backward-delta distribution. Finally, the full posterior distribution that shows the possible accumulation values that start at the initial condition and result in the appropriate choice or sign of the accumulation value. In this example trial we consider a trial where there is change in state at 750ms, and we evaluate a left choice ($a < B$) at $t = 1$s. The entire solution was computed using 51 backward-delta solutions on a grid from $(-50, B)$ with $\Delta a = 1$. We can also examine slices through the posterior distribution at various time points to confirm agreement between the trajectories and the analytical solution (Fig. 9).

A few notes on the advantages of the analytical solution. First, the analytical model offers a large increase in accuracy of the model over previous numerical approaches. Second, the analytical model is much faster to fit and evaluate. Second, we can compute the posterior

distribution for only a subset of all time points without computing the solution for all time points. This fact allows for very rapid computation of the posterior distribution.

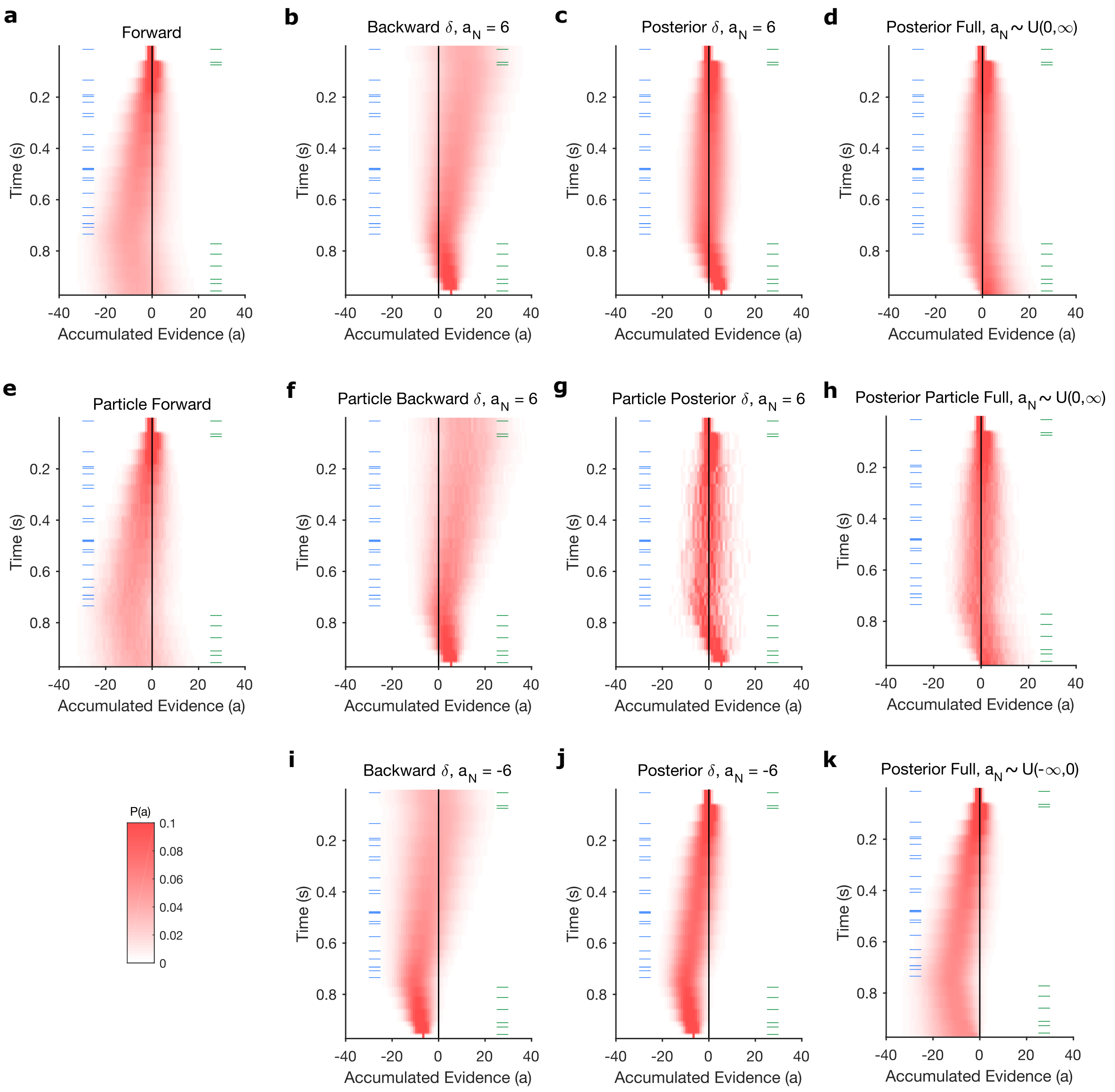

Supplementary Figure 8: **Full trial model posterior validation** Comparison of the model distributions computed from the analytical solution (top and bottom) and sampled trajectories (middle) for an example trial. Green ticks mark times of right clicks, blue ticks mark times of left clicks. (A, E) Forward distribution assuming $a_0 = 0$. (B, F) Backward-delta distribution assuming a final accumulation value of $a_n = 6$. (C, G) Posterior-delta distribution assuming $a_n = 6$ and $a_0 = 0$. (D, H) Full posterior distribution assuming $a_0 = 0$ and a right choice ($a > 0$). (I) Backward-delta distribution assuming a final accumulation value of $a_n = -6$. (J) Posterior-delta distribution assuming $a_n = 6$ and $a_0 = 0$. (K) Full posterior distribution assuming $a_0 = 0$ and a left choice ($a < 0$). Source data are provided as a Source Data file.

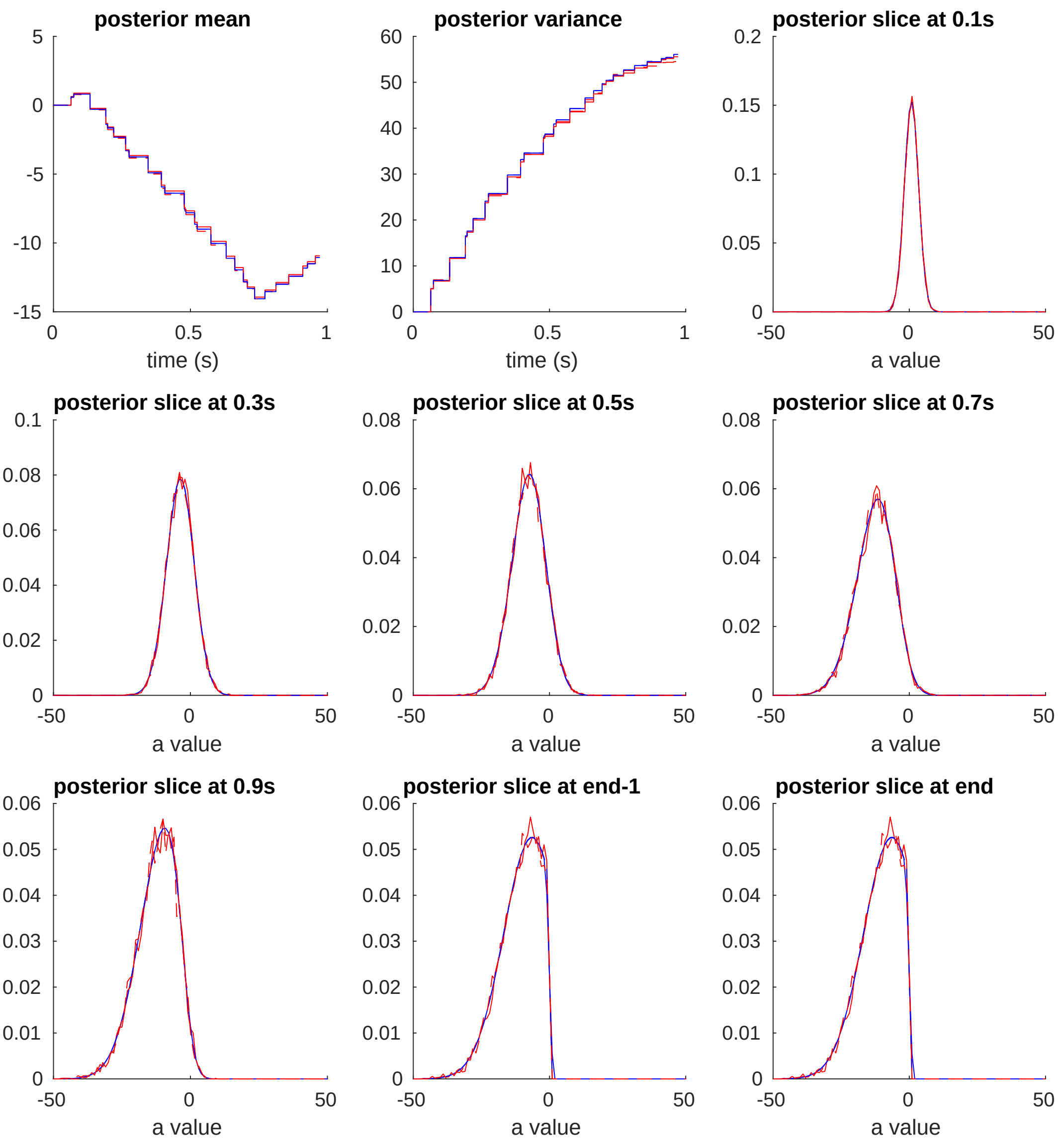

Supplementary Figure 9: **Within trial model posterior validation** Comparison of the posterior distribution computed from the analytical solution (blue) and sampled trajectories (red) for an example trial for the mean and variance over time as well as for slices at specific timepoints. This figure shows the same distribution as Fig. 8K (Red). Source data are provided as a Source Data file.

4 Accumulation value tuning curves

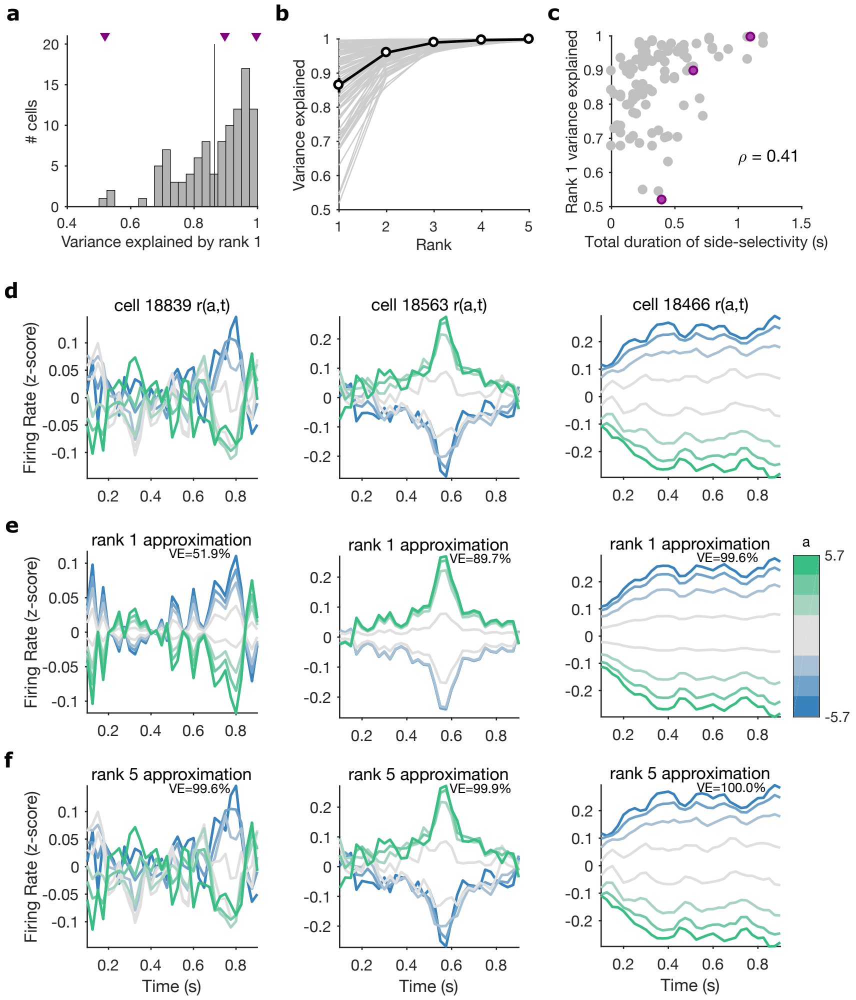

Supplementary Figure 10: **Rank 1 decomposition variance explained for all side-selective cells** Comparison of variance explained by the rank 1 decomposition for side-selective cells. (A) Histogram of variance explained by rank 1 decomposition for all side-selective cells. Black vertical line marks the mean variance explained and purple arrows mark variance explained for the lowest, middle, and highest variance explained cells. (B) Variance explained as a function of the rank of the decomposition for all cell (gray traces). Black trace marks the mean and errorbars represent 95% confidence intervals. (C) Rank 1 variance explained as a function of the total duration of side-selectivity (significant AUC favoring for the cell's preferred side). These values are positively correlated (Pearson's correlation, $\rho = 0.41, p < .01$). (D) Residual z-score firing rate map for example cells for the cell's with the least (left), most (right), and the middle (middle) variance explained by the rank 1 decomposition. (E) Rank 1 approximation for the cells in B with annotations showing the variance explained (VE). (F) Rank 5 approximations, plotted as in C. Source data are provided as a Source Data file.

5 Supplementary Note 1

Model State Changes

We predicted the timing of changes of mind using the time points within a trial when the mean of the posterior distribution $p(a) = P(a|t, \delta_R, \delta_L, \theta, a_0 \sim \mathcal{N}(0, \sigma_i^2), y)$ crossed the decision boundary B. These trajectories have several sources of noise that complicated our analysis. First, they have sharp discontinuities at the times of each stimulus click. This sometimes resulted in the mean trajectory repeatedly crossing the decision boundary in a short period of time. It is unlikely that each of these crossings corresponded to a true change of mind, since the subject was likely in a general state of indecision (Fig. 11A). To resolve this issue we smoothed the mean trajectories with a 100 ms running average. This smoothing resolved the flickering model state changes from individual trials (Fig. 11B). However, there were still model state changes that only briefly crossed or oscillated around the decision boundary.To detect and remove these time points we estimated the local slope of the smoothed trajectory, and filtered out model state changes where the local slope had an inconsistent sign with the direction of the state change (Fig. 11C). We excluded any state change that occurred in the first or last 200ms of the trial.

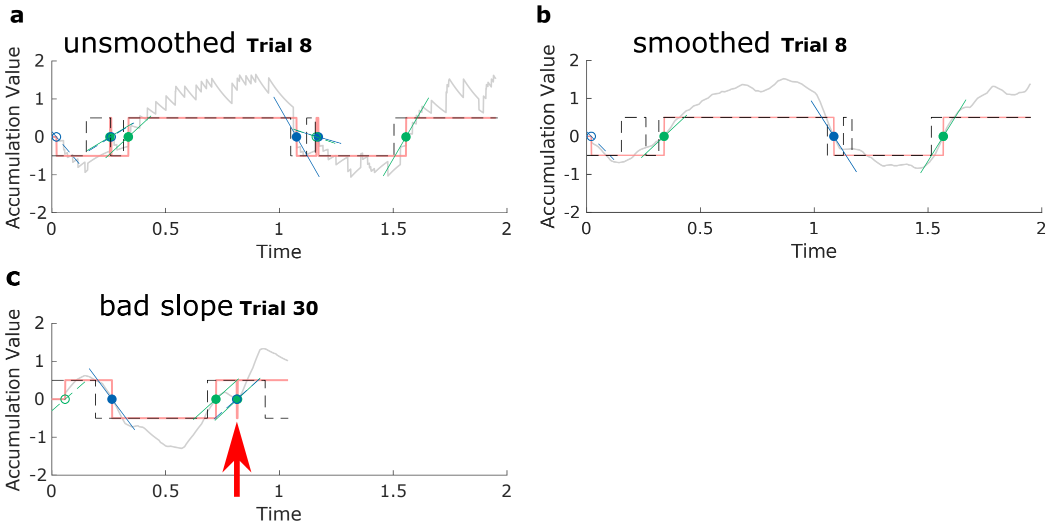

Supplementary Figure 11: **Analysis of smoothing model mean and weak and strong state changes** (A) The mean trajectory of the model posterior is shown in gray, along with the categorical decision taken from the sign of the model accumulation value in red. The generative state (dashed black traces) is overlaid on the model state indicator. Each model state change is marked with a circle. Model state changes to the "go right" state are in green, and changes to the "go left" state in blue. The local slope is marked with a colored line. The initial decision at the start of the trial was excluded (empty circle). (B) The mean trajectory was smoothed, removing spurious changes when the trajectory flickered near the decision boundary. (C) In this example trial around 750ms, the mean trajectory is generally moving to the "go right" (positive accumulation value) state, but briefly returns to the go-left state. This creates a situation where there are multiple model state changes at the same time, with an incongruent slope (red arrow). Source data are provided as a Source Data file.

Supplementary References

[1] Brunton, B. W., Botvinick, M. M., and Brody, C. D. (2013). Rats and humans can optimally accumulate evidence for decision-making. *Science*, 340(6128):95–98.

[2] Piet, A. T., El Hady, A., and Brody, C. D. (2018). Rats adopt the optimal timescale for evidence integration in a dynamic environment. *Nature Communications*, 9(1):4265.
