# Supplementary material for: Stable choice coding in rat frontal orienting fields across model-predicted changes of mind
Source: Nat Commun. 2022 Jun 10;13:3235. doi: 10.1038/s41467-022-30736-3 (PMC9187710; doi:10.1038/s41467-022-30736-3)
Supplement: Supplementary file 3 — Reporting Summary [file 41467_2022_30736_MOESM3_ESM.pdf]

nature research

Corresponding author(s): Carlos Brody

Last updated by author(s): Mar 2, 2022

Reporting Summary

Nature Research wishes to improve the reproducibility of the work that we publish. This form provides structure for consistency and transparency in reporting. For further information on Nature Research policies, see Authors & Referees and the Editorial Policy Checklist .

Statistics

For all statistical analyses, confirm that the following items are present in the figure legend, table legend, main text, or Methods section.

n/a	Confirmed	
☐	☒	The exact sample size (*n*) for each experimental group/condition, given as a discrete number and unit of measurement
☐	☒	A statement on whether measurements were taken from distinct samples or whether the same sample was measured repeatedly
☐	☒	The statistical test(s) used AND whether they are one- or two-sided<br>*Only common tests should be described solely by name; describe more complex techniques in the Methods section.*
☒	☐	A description of all covariates tested
☐	☒	A description of any assumptions or corrections, such as tests of normality and adjustment for multiple comparisons
☐	☒	A full description of the statistical parameters including central tendency (e.g. means) or other basic estimates (e.g. regression coefficient) AND variation (e.g. standard deviation) or associated estimates of uncertainty (e.g. confidence intervals)
☐	☒	For null hypothesis testing, the test statistic (e.g. *F*, *t*, *r*) with confidence intervals, effect sizes, degrees of freedom and *P* value noted<br>*Give P values as exact values whenever suitable.*
☐	☒	For Bayesian analysis, information on the choice of priors and Markov chain Monte Carlo settings
☒	☐	For hierarchical and complex designs, identification of the appropriate level for tests and full reporting of outcomes
☐	☒	Estimates of effect sizes (e.g. Cohen's *d*, Pearson's *r*), indicating how they were calculated

Our web collection on statistics for biologists contains articles on many of the points above.

Software and code

Policy information about availability of computer code

Data collection	We used custom MATLAB code to train the rats to perform the task. This code is available upon reasonable request via email to the correspondence author. MATLAB version 2013b was used to train the rats. The training code, including the anti-biasing algorithm can be referred to as version 1 in requests for code sharing. The code is version controlled and maintained on github, so we can provide the code as used in this study. However, we will keep the training code in a private repository.<br><br>We used Cheetah and Trodes for electrophysiological data acquisition using arrays and tetrodes, respectively. We used SpikeSort3D to isolate units from array recordings and Kilosort2 and the Phy GUI to isolate units from tetrode recordings.
Data analysis	The custom MATLAB and julia code used to perform all analysis is available at https://github.com/Brody-Lab/dynamic_ephys.<br>For analysis, we used MATLAB version 2016b and julia version 1.2.0.

For manuscripts utilizing custom algorithms or software that are central to the research but not yet described in published literature, software must be made available to editors/reviewers. We strongly encourage code deposition in a community repository (e.g. GitHub). See the Nature Research guidelines for submitting code & software for further information.

Data

Policy information about availability of data

All manuscripts must include a data availability statement. This statement should provide the following information, where applicable:

- Accession codes, unique identifiers, or web links for publicly available datasets
- A list of figures that have associated raw data
- A description of any restrictions on data availability

The datasets generated and analyzed in this study are available at https://figshare.com/articles/dataset/Manuscript_Data/16695592. These data are sufficient to reproduce all figures in the study. In addition, we provide source data files for each figure.

Field-specific reporting

Please select the one below that is the best fit for your research. If you are not sure, read the appropriate sections before making your selection.

☒ Life sciences ☐ Behavioural & social sciences ☐ Ecological, evolutionary & environmental sciences

For a reference copy of the document with all sections, see nature.com/documents/nr-reporting-summary-flat.pdf

Life sciences study design

All studies must disclose on these points even when the disclosure is negative.

Sample size	Sample sizes were determined by the limits of what data could be collected within a reasonable timeframe and standards of the field. Five adult male Long-Evans rats (Hilltop, Harlan and Taconic) were used for the experiments presented in this study. All were implanted with recording probes. Four of these were unilateral electrode arrays (2 left, 2 right) and one was a bilateral tetrode drive.
Data exclusions	For electrophysiological analyses, we recorded and isolated 738 cells We excluded cells with a firing rate of less than 1 Hz during the task, consistent with standard analyses in the field, giving us 592 cells. Where noted, we further limited analyses to the 103 cells that met a pre-determined pre-movement side-selectivity metric, consistent with a previous paper Hanks et al., 2015.<br><br>We restricted behavioral analysis to sessions where overall accuracy exceeded 70%, the center fixation violation rate was below 25%, and rats performed more than 50 trials. These exclusions were predetermined and consistent with previous studies.
Replication	We did not attempt to replicate the effects observed across the 5 independently collected rat datasets presented here.
Randomization	The only experimental condition that varied across subjects was the hemisphere selected for probe implantation. This condition was counterbalanced and randomly assigned.
Blinding	Our study did not consist of comparisons between groups that would have required blinding, therefore investigators were not blinded to information about subjects used in the study.

Reporting for specific materials, systems and methods

We require information from authors about some types of materials, experimental systems and methods used in many studies. Here, indicate whether each material, system or method listed is relevant to your study. If you are not sure if a list item applies to your research, read the appropriate section before selecting a response.

Materials & experimental systems

n/a	Involved in the study	
☒	☐	Antibodies
☒	☐	Eukaryotic cell lines
☒	☐	Palaeontology
☐	☒	Animals and other organisms
☒	☐	Human research participants
☒	☐	Clinical data

Methods

n/a	Involved in the study	
☒	☐	ChIP-seq
☒	☐	Flow cytometry
☒	☐	MRI-based neuroimaging

Animals and other organisms

Policy information about studies involving animals; ARRIVE guidelines recommended for reporting animal research

Laboratory animals	Male Long–Evans rats (Rattus norvegicus) were used for this study. Data was collected from H037 between ages of 8 and 20 months, from H066 between 11 and 27 months, from H084 between 6 and 18 months, from H129 between ages 10 and 15 months and from H191 between ages 6 and 21 months.
Wild animals	The study did not involve wild animals.
Field-collected samples	The study did not involve the Field-collected samples from the filed.
Ethics oversight	All animal use procedures were approved by the Princeton University Institutional Animal Care and Use Committee and carried out in accordance with NIH standards.

Note that full information on the approval of the study protocol must also be provided in the manuscript.
